# Evolutionary Origin and Genetic Diversity of the Pannonian Ecotype of *Apis mellifera carnica* Colonies in Hungary Based on Mitochondrial DNA and Microsatellite Markers

**DOI:** 10.3390/biology14050475

**Published:** 2025-04-25

**Authors:** Reka Balazs, Tamas Gergely Molnar, Erika Edvine Meleg, Andras Hidas, Edit Zajacz, Timea Racz, Nora Palinkas-Bodzsar

**Affiliations:** 1 Institute for Farm Animal Gene Conservation, National Centre for Biodiversity and Gene Conservation, 2100 Godollo, Hungary; molnar.tamas.gergely@uni-mate.hu (T.G.M.); meleg.erika@nbgk.hu (E.E.M.); hidas.andras@nbgk.hu (A.H.); zajacz.edit@nbgk.hu (E.Z.); racz.timea@nbgk.hu (T.R.); palinkasbodzsar.nora@nbgk.hu (N.P.-B.); 2 Doctoral School of Animal Biotechnology and Animal Science, Hungarian University of Agriculture and Life Sciences, 2100 Godollo, Hungary; 3 Department of Applied Fish Biology, Institute of Aquaculture and Environmental Safety, Hungarian University of Agriculture and Life Sciences, 7400 Kaposvar, Hungary

**Keywords:** honey bee, morphological breed standard, mitochondrial DNA, microsatellite, genetic conservation

## Abstract

Honey bees are crucial to both the ecosystem and the economy. However, they are subject to different influences that can lead to a loss of genetic diversity. In this study, we used mitochondrial DNA information and nuclear microsatellite markers to compare worker individuals that strictly meet the morphological breed standard of the Pannonian bee in Hungary to those with morphological disorders (yellow color of the abdomen). Additionally, this study involves Carniolan colonies from two European countries and other bee subspecies as a reference group that might have crossed into the Pannonian bee. As for the mitochondrial DNA, the combined assessment of *COI* and *16S* genes identified six haplotypes. Based on the tRNAleu-cox2 intergenic region (E2/H2), our samples belonged to the C evolutionary lineage. According to the microsatellite data, the level of inbreeding was low in all groups investigated, and only the genotypes of the Pannonian bee showed significant deviation from the Hardy–Weinberg equilibrium state. Cluster analysis and the Discriminant Analysis of Principal Components showed that bees that failed the morphological breed identification had started to diverge genetically from those meeting the breed standards, becoming more similar to the Carniolan bee. Our findings suggest that the genetic status of the Pannonian bees investigated in this study is satisfactory. However, in order to maintain an adequate level of diversity, periodic genetic monitoring of the colonies is necessary.

## Simple Summary

Honey bees are essential for both nature and agriculture. However, their genetic diversity may decline due to environmental and human influences. This study reveals the genetic background of the Pannonian bee, the only officially recognized bee breed in Hungary. Individuals that meet the breed standard were compared to those showing morphological disorders (yellow color of the abdomen). Additionally, we investigated Carniolan bees from two other European countries and other subspecies that may have hybridized with the Hungarian native bee in the past. Our results indicated that the Pannonian bee belongs to the C evolutionary lineage, which represents the North Mediterranean subspecies. They showed a low level of inbreeding with an appropriate level of genetic diversity, suggesting stable bee groups. Furthermore, bees with morphological defects are gradually becoming genetically distinct from the standard Pannonian bee and more similar to the Carniolan bees. These findings help to confirm the breed identification of the Pannonian bee and provide useful information for beekeeping and genetic conservation, ensuring the long-term sustainability of honey bee populations.

## 1. Introduction

Honey bees have an important role both in ecosystems and the economy [1]. Currently, more than 30 subspecies of *Apis mellifera* can be identified based on their morphological, geographical, genetic, and behavioral characteristics [2,3]. In many countries and regions—e.g., Europe, the USA, and near the Arctic Circle—the decline of wild and domestic honey bee populations is a major problem [4,5,6,7]. The ongoing population decrease can be attributed to various diseases [8], the use of harmful insecticides [9], the loss of biodiversity in flora [10], and the introduction of invasive subspecies [11,12]. Several gene conservation programs have been established in many European countries, such as Denmark and France [13,14], to preserve their native honey bee populations [15].

In honey bees, haploid individuals have 16 chromosomes, and their genome length is ~250 Mbp [16,17]. Their mitochondrial genome is approximately 16,000–17,000 base pairs in length, inherited on the maternal side and relatively free of recombination, therefore suitable for origin studies [18,19,20]. It is possible to determine the origin of populations and their migration routes [18] by mitochondrial DNA (mtDNA) markers, as genetic exploration has been conducted for several subspecies (e.g., *Apis mellifera caucasica*, *Apis mellifera syriaca*) [21,22], allowing comparative studies to be carried out. Based on mitochondrial DNA analysis, the subspecies of *Apis mellifera* have been classified into six different evolutionary lineages. Lineage A primarily includes African subspecies, while lineage C consists of North Mediterranean subspecies, and lineage M represents the West and North European subspecies [23]. Lineage O is associated with the Near Eastern subspecies [24] while lineage Y is associated with the Ethiopian subspecies [25]. The S/Z lineage comprises Syrian, Lebanese, and Iraqi honey bee populations [26,27].

The Carniolan bee (*Apis mellifera carnica*) belongs to the C evolutionary lineage [28] and is one of the most popular commercial subspecies because of its calmer and gentler behavior compared to other bees, such as *Apis mellifera mellifera* [2,29]. Two ecotypes of the Carniolan bee have been identified: the Alpine (Austria, Slovenia) and the Pannonian (Hungary, Croatia, Romania). There are both morphological [2,30] and genetic differences between these ecotypes [31,32]. There are differences between the ecotypes in the honey-gathering period, honey production, and overwintering ability [33]. The Pannonian bee in Hungary is well adapted to the local environment, overwinters well with low honey consumption, develops rapidly in spring, and has excellent honey production [34]. The Pannonian bee is the only one that is allowed to be bred in Hungary as an officially recognized bee breed by the Animal Breeding Directorate of the National Food Chain Safety Office, according to Decision No. 02.5/2297-2/2012, effective from 21 August 2012 [34,35]. The breeding is managed by the Hungarian Bee-Breeders’ Association (HBBA), which is authorized to register the Pannonian bee breed and issue certificates of origin. The members of the HBBA operate state-licensed queen bee breeding apiaries in Hungary [34]. Since 1976, each year, the HBBA commissions the Department of Apiculture and Bee Biology of our institute to perform regular checks on the morphological characteristics (color pattern, proboscis length, and cubital index) of reared bee colonies. Based on these assessments, bee breeders can obtain a breeding license. The color pattern of the abdomen of the Pannonian bee is dark, but it is acceptable if a yellow spot appears on both sides of the first and second tergites, provided that it does not exceed 20% of the colony. If the yellow color is present to a greater extent, it is considered a defect. If yellow color appears on the third and fourth tergites, the bee colony immediately fails the morphological verification, and the queen is excluded from breeding. The accepted length of the proboscis in the Pannonian bee is between 6.50 and 6.82 mm, while the cubital index ranges between 2.3 and 3.2 [34]. The morphological qualification tests are used to support the maintenance of purebred colonies and gene conservation in order to prevent further reproduction of individuals with foreign genetic material. Despite these efforts, a local study has detected the presence of the Italian bee (*Apis mellifera ligustica*, 2.5%), Buckfast hybrid (1.7%), and European dark bee (*Apis mellifera mellifera*, 2.2%) in Hungarian honey bee samples [36].

The first objective of the present study was to determine the evolutionary origin and diversity of the Pannonian bee colonies reared in Hungary using mitochondrial DNA information. A further aim was to assess their nuclear genetic diversity based on microsatellite marker analysis. The question was the extent of genetic difference between the Pannonian bee in Hungary that met the breed standard and the morphologically defective individuals from colonies that failed the morphological breed identification. Moreover, comparisons were performed with Carniolan bees from other European countries and with a group of other honey bee subspecies, including *Apis mellifera ligustica*, Buckfast hybrids, and Buckfast × Italian hybrids.

From these results, we can conclude whether the breeding and morphological breed identification methods used in Hungary for gene conservation are appropriate or require improvement. This may provide useful information for bee breeding and conservation programs for local bees in other countries as well.

## 2. Materials and Methods

According to Government Decree 40/2013 (II. 14.), an animal testing permit is not required to conduct experiments on honey bees.

### 2.1. Sample Collection

The honey bee individuals were sent to our institute by the Pannonian bee breeders in early autumn for the official annual morphological breed identification (color, proboscis length, and cubital index). These samples were received with the breeders’ permission for further genetic investigations while preserving their anonymity.

A total of 144 samples were collected from adult worker bees and were grouped into four categories (Table S1). The MF group consisted of 64 individuals from Pannonian bee colonies that met the morphological breed standard over a period of 3 years based on the officially performed breed identification. These samples came from 16 apiaries, well-distributed across Hungary and covering the major beekeeping regions. Four colonies were selected per beekeeper, with one sample per colony. Colonies that failed the official morphological assessment criteria were chosen for the NMF group. These individuals exhibited one or two yellow tergites. Since colonies that do not meet the breed standard are relatively rare, we were able to select 8 apiaries, with only one colony per beekeeper and 4 individuals per colony, in a total of 32 samples. Considering a previous study [36] where the presence of the above-mentioned subspecies was found, a reference group (REF) was established. This included 32 samples: 16 individuals from 4 Buckfast hybrid colonies (4 samples per colony), 8 worker bees from an Italian bee (*Apis mellifera ligustica*), and 8 from a Buckfast × Italian hybrid colony. Finally, 16 Carniolan bees (*Apis mellifera carnica*) samples were collected from two colonies (8 samples per colony) of two other European countries (Ukraine and Slovenia: 1 colony per country) for the fourth group (KRAJ). As the Pannonian bee is basically derived from this subspecies, we aimed to explore the actual genetic relationship between them.

### 2.2. DNA Extraction

All 144 samples were analyzed with mitochondrial DNA and microsatellite markers, 64 samples of the MF, 32 of the NMF, 32 of the REF, and 16 of the KRAJ groups (Table S1). DNA samples were extracted using a modified method described by Latorre et al. [37]. The last two legs and the abdomen were removed from the body and then mechanically disrupted. A total of 160 µL of Buffer I (100 mL of 0.12 g Tris-HCl, 2 mL of EDTA solution (pH 8), 0.35 g of NaCl, and 5 g of sucrose (pH 7.8)) was added to each sample in a 1.5 mL Eppendorf tube and then vortexed. The next step was to add 100 µL of Buffer II (100 mL of 3.63 g Tris-HCl, 2 mL EDTA solution (pH 8), 1.25 g SDS (Sodium lauryl sulfate), and 5 g sucrose (pH 9)), followed by vortexing. Afterward, the mixture was incubated at 65 °C for 1 h, and 60 µL of 3 M sodium-acetate was added, followed by vortexing. The mixture was incubated at room temperature for 30 min and centrifuged at 13,000 rpm for 15 min. Subsequently, 300 µL of the supernatant was mixed with 300 µL of isopropanol in a new 0.6 mL Eppendorf tube and left at room temperature for 5 min, then turned over. The mixture was centrifuged at 13,000 rpm for 10 min. The supernatant was discarded, and 500 µL of 70% ethanol was added to each tube. The tubes were then centrifuged at 13,000 rpm for 5 min, and this step was repeated twice. After the final wash, all liquid was removed from the tube, and the sample was dried for 1 h. After drying, 60 µL of TE (TRIS-EDTA) buffer was added to the DNA and incubated overnight at 37 °C. DNA quantity and quality were measured by NanoDrop 2000c (Thermo Fisher Scientific, Waltham, MA, USA) and then equalized to 30 ng/µL for mitochondrial DNA and 6 ng/µL for microsatellite analysis.

### 2.3. Mitochondrial DNA

Three different regions of the mtDNA were examined in this study. The cytochrome oxidase I (*COI*) and the rRNA (*16S*) genes were used for assessing the mitochondrial genetic variance, while the tRNAleu-cox2 (*COI-COII*) intergenic region (E2/H2) was used for determining the evolutionary origin, lineages. The primer sequences were as follows: *COI* primer forward (5′-CTGATATAGCATTCCCCCGAATA-3′) and reverse (5′-AGAATTGGATCTCCACGTCCTA-3′) [36]; *16S* primer forward (5′-ACATCGAGGTCGCAAACATC-3′) and reverse (5′-TTAGGTCGATCTGCTCAATGAA-3′) [38]; and for the *COI-COII* intergenic region, the E2 forward (5′-GGCAGAATAAGTGCATTG-3′) and H2 reverse (5′-CAATATCATTGATGACC-3′) primers were used [23,39].

The total volume of the PCR was 20 µL, which contained 150 ng/µL of genomic DNA, 10× Dream Taq buffer with 20 mM MgCl_2_ content (Thermo Fisher Scientific, Waltham, MA, USA), 5 µM of each primer, 0.2 mM dNTP mix (Thermo Fisher Scientific, Waltham, MA, USA), and Dream Taq DNA Polymerase (1 unit) (Thermo Fisher Scientific, Waltham, MA, USA). The PCR reaction consisted of an initial denaturation step at 94 °C for 2 min, followed by 35 cycles. Each cycle included 45 s at 94 °C, 45 s at 46 °C (*COI*, E2/H2) or 48 °C (*16S*), and 45 s at 72 °C. The final elongation was at 72 °C for 5 min. The PCR products were stored at −20 °C until sequencing. The purification of the PCR products and the Sanger sequencing of the samples were performed by BIOMI Ltd. (Godollo, Hungary) as part of their service for all three mtDNA regions investigated.

### 2.4. Microsatellite Genotyping

In total the same 144 samples were genotyped using 20 microsatellite markers, including A29, A35, A88, A107, A113 [40], A007, A(B)24, Ac011, Ac306, Ap049, Ap218, Ap226, Ap289, Ap307 [41], Ap033, Ap043 [42], A008 [43], Ap055, Ap066, and Ap081 [44]. The markers were selected from the literature based on their polymorphism information content (PIC), which fell into the fairly or very informative category. They had been tested previously on Pannonian bee colonies (preliminary investigations) and, in an earlier study, on Hungarian bees [36]. To reduce the time and cost of the investigation, tailed primers were used for genotyping, using an 18 bp long sequence (5′-CAGGACCAGGCTACCGTG-3′) inserted at the 5′ end of the forward primers [45]. The tail sequence (universal primer) was labelled with 3 different fluorescent dyes (WELL-RED) (D2: black, D3: green, D4: blue). Thus, the marker sets were optimized by multiplexing within PCR reactions (6 multiplex, 3 simplex) and/or pooling PCR products. The fragment analysis was organized into 3 sets based on the combinability of different allele sizes. Therefore, the genotyping was performed with 6–7 markers at the same time, which was also an important factor in selecting the markers (Table S2).

For each sample, the final volume of 15 µL master mix contained 10× Dream Taq Buffer with 20 mM MgCl_2_ (Thermo Fisher Scientific, Waltham, MA, USA), 5 μM of each primer, 0.2 mM dNTP mix (Thermo Fisher Scientific, Waltham, MA, USA), Dream Taq DNA Polymerase (1 unit) (Thermo Fisher Scientific, Waltham, MA, USA) and 30 ng genomic DNA. PCR products were detected by capillary gel electrophoresis using an automated DNA sequencer (GenomeLab™ GeXP Genetic Analysis System, Beckman Coulter, Inc., 4300 North Harbor Boulevard, Fullerton, CA, USA). The allele sizes were determined using a 400 bp allele ladder (GenomeLab™ DNA Size Standard-400, Beckman Coulter, Inc., 4300 North Harbor Boulevard, Fullerton, CA, USA) according to the manufacturer’s instructions.

### 2.5. Statistical Methods Used for Data Analysis

#### 2.5.1. Mitochondrial DNA Analysis

The quality of the raw sequences was checked very carefully before the analysis. The *COI* and *16S* genes were evaluated together; synthetic sequences were generated by the alignment of the sequences obtained from the two mtDNA regions of the same sample. As the *COI-COII* intergenic region (E2/H2) was used for determining the evolutionary lineages, it was assessed separately from the *COI* and *16S* genes. The sequences were aligned using the MEGA11 11.0.13 software [46] and analyzed with the ClustalW algorithm [47]. Subsequently, haplotypes and diversity indices were determined, such as the nucleotide diversity (π), haplotype diversity (Hd), Fu’s Fs value, Tajima’s genetic distance (D) and their significance level. These values were calculated for each honey bee group (MF, NMF, REF, and KRAJ) using DnaSP v6.12.03 software (DNA Sequence Polymorphism v6.12.03, [48]). The Tajima’s D value was determined by the number of segregating sites and the average number of nucleotide differences from pairwise comparisons. This value allows us to infer the genetic variability equilibrium within the population. A positive value indicates an excess of intermediate-frequency polymorphisms, while a negative value may suggest an excess of low-frequency polymorphisms [49]. Fu’s Fs value is based on the frequency of haplotypes and can infer processes in the population, such as logistic growth or background selection by the evolution of a locus. A negative value may indicate an excess number of alleles compared to what is expected under neutrality, while a positive value may suggest a reduction in these alleles [50]. Our haplotypes were compared to sequences available in the NCBI (National Center for Biotechnology Information) database by the nucleotide BLAST (Basic Local Alignment Search Tool, https://blast.ncbi.nlm.nih.gov/Blast.cgi (accessed on 21 April 2025)) program [51] to assess their uniqueness. The network of the haplotypes was built using the median-joining algorithm of Network 10 software [52] based on the genetic distance between them. In the case of the *COI* and *16S* genes, 45 sequences of different *Apis mellifera* subspecies from the NCBI database were involved. For the intergenic *COI-COII* region (E2/H2) 62 *Apis mellifera* sequences of the NCBI database were used to more accurately describe the evolutionary origin of the Pannonian bee. For visualization of the evolutionary lineages, a maximum likelihood phylogenetic tree was constructed as well, using MEGA 11 11.0.13 software with the model of Tamura 3-parameter [46].

#### 2.5.2. Microsatellite Marker Analysis

The results for the microsatellite markers were analyzed using the GenomeLab Genetic Analysis System 10.2 software (Beckman Coulter, Inc., 4300 North Harbor Boulevard, Fullerton, CA, USA). Genetic diversity parameters, such as the mean number of alleles (MNA), unbiased expected (H_E_) and observed (H_O_) heterozygosity, and inbreeding coefficients (F_IS_) were calculated by the Microsatellite Toolkit program [53]. The level of population differentiation was estimated by the Analysis of Molecular Variance (AMOVA) using GenAlEx 6.5 software [54,55].

Discriminant Analysis of Principal Components (DAPC) using microsatellite loci and populations was performed in the R environment (4.2.1) with the adegenet 2.1.1.7 package [56]. The software evaluated the a-score for different numbers of PCs, and the optimal number of 15 was selected that balanced discrimination power and overfitting. Cluster analysis was performed using a Bayesian-based approach with the STRUCTURE 2.3.4 software [57]. This software categorizes individuals into groups based on their allele patterns without any previous information regarding their population affiliation. The analysis was carried out with an admixture model with correlated allele frequencies. It was run with 20,000 burn-in periods, followed by 50,000 iterations for each K number ranging from 2 to 6. For each K value, 100 independent runs were performed. Pairwise comparisons of the 100 solutions for each K value were made in a greedy algorithm. Clusters with the highest average similarity index were visualized using the CLUMPAK 1.1 software package [58], as well as the most likely clustering (delta K) based on the Evanno method [59].

## 3. Results

### 3.1. Mitochondrial DNA Information of the Honey Bee Groups Studied

#### 3.1.1. Genetic Variance of the Pannonian Bee Based on the COI and 16S mtDNA Regions

In case of the *COI* gene, seven polymorphic sites (Figure S1) were identified, and the 142 sequences (352 bp length, GenBank Acc. no.: PQ686393—PQ686534) could be classified into five haplotypes (Table S3). None of these were found in the NCBI database, indicating that they can be considered new and unique. In comparison to the reference *Apis mellifera* mitochondrial genome (GenBank Acc. no.: MN250878.1), our sequenced *COI* gene corresponded to nucleotides starting at position 2116. For two haplotypes, the polymorphism resulted in changes in the amino acids. Compared with the sequence from the NCBI database (GenBank Acc. no.: NC_051932.1, Gene ID: 60455728), the H4 haplotype had methionine (MET) at position 180 instead of isoleucine (Figure S2), while the H3 haplotype had isoleucine at position 181 instead of valine (Figure S3).

The sequence of the *16S* gene is a 345 bp segment (GenBank Acc. no.: PQ721124—PQ721264) starting at 13,560 bases in reference sequence (GenBank Acc. no.: MN250878.1). Four polymorphic sites were detected (Figure S4), and the samples (*n* = 141) were categorized into four haplotypes based on them, two of which were not found in the NCBI database (Table S4).

The sequences obtained from the 141 samples of *COI* and *16S* genes were merged into one synthetic sequence for each individual. The first nucleotide of the 635 bp long synthetic sequence corresponds to the first bp of the *COI* sequence, while the 353. bp is the first nucleotide of the *16S* gene sequence. These sequences were classified into six haplotypes (Table 1).

The major haplotype was the H1, which included 75.2% of the samples containing individuals from all four groups investigated. This haplotype includes individuals not only of Pannonian bees but also from the REF group, such as *Apis mellifera ligustica*, the Buckfast hybrid, and the Buckfast × Italian hybrid too. Within haplotypes, the total number of polymorphic sites was ten. In comparison to the H1 haplotype, the H6 haplotype is characterized by five base substitutions, with this group encompassing individuals of the Buckfast hybrid. The MF group included four haplotypes. Only 1–2 individuals were associated with the H3 and H4 haplotypes (Table 1).

Estimates of genetic variance were calculated individually for the *COI* and the *16S* genes (Tables S5 and S6) as well as jointly (Table 2). The highest haplotype diversity (Hd) was found in the KRAJ group (0.525 ± 0.055), while the MF stock had the lowest. The nucleotide diversity was lowest in the MF group (π = 0.00032) and highest in the REF group (π = 0.00278). Both the Tajima’s D-value and Fu’s Fs value were negative in the MF group, which may indicate an increase in its population size or positive selection. In contrast, they were positive in the NMF, REF, and KRAJ groups, showing a decrease in population size or balancing selection.

**Figure 1:**
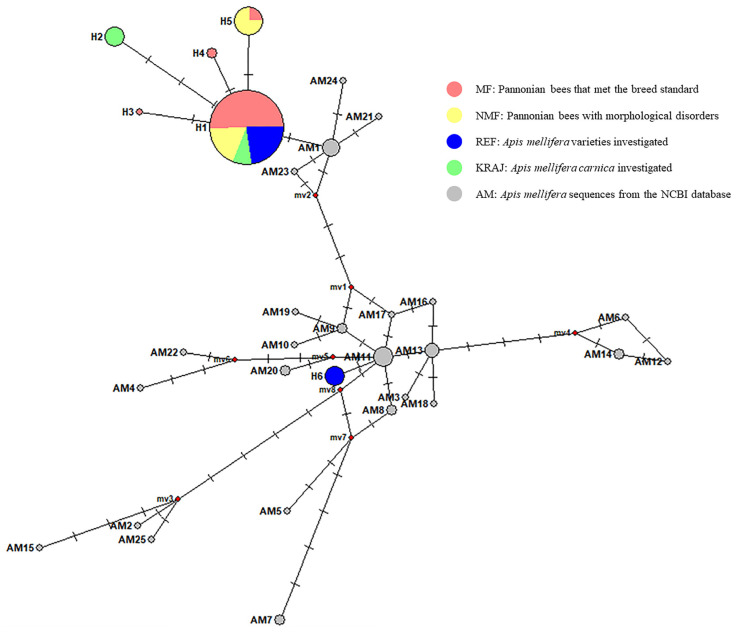
Median-joining network illustrating the relationships of haplotypes obtained from the joint analysis of *COI* and *16S* genes. H1–6—haplotypes containing our samples, AM1-25—haplotypes containing sequences of the NCBI database (Table S7). The size of each circle is directly proportional to the frequency of the corresponding haplotypes. The median vector (mv is produced by the network software) represents a putative intermediate haplotype that has not been observed in the samples investigated. GenBank accession numbers of the sequences belonging to the AM haplotypes: AM1—AP018403.1, AP018404.1, AP018432.1, KY464957.1, MH341407.1, MN250878.1; AM2—CM040891.1; AM3—KJ396182.1; AM4—KJ396183.1; AM5—KJ396184.1; AM6—KJ396185.1; AM7—KJ396186.1, MN119925.1; AM8—KJ396187.1, MG552699.1; AM9—KJ396188.1, KY614238.1; AM10—KJ396189.1; AM11—KM458618.1, MG552682.1, MG552694.1, MG552697.1, MG552701.1, MN585110.1, MN714162.1, MZ981768.1; AM12—KP163643.1; AM13—KX870183.1, KX943034.1, MG552681.1, MG552698.1; AM14—KY464958.1, MN714161.1; AM15—KY926884.1; AM16—MF678581.1; AM17—MG552687.1; AM18—MG552692.1; AM19—MG552693.1; AM20—MG552703.1, MN585109.1; AM21—MH341408.1; AM22—MN585108.1; AM23—MT188686.1; AM24—MW811175.1; and AM25—OK075087.1.

The sequences from the joint analysis of the *COI* and *16S* genes of the honey bees studied were compared with 45 *Apis mellifera* sequences of other bee subspecies from the NCBI database using the NETWORK 10 software (Table S7, Figure 1). None of the sequences from the NCBI database showed 100% identity with the samples studied. However, H1, H2, H3, H4, and H5 showed high similarity to the following subspecies: *Apis mellifera ligustica* (GenBank Acc. no.: MH341408.1), *Apis mellifera carnica* (GenBank Acc. no.: MW811175.1), Buckfast hybrid (GenBank Acc. no.: AP018432.1), and *Apis mellifera caucasica* (GenBank Acc. no.: AP018404.1). The H6 haplotype demonstrated similarity to *Apis mellifera capensis* (GenBank Acc. no.: MG552682.1), *Apis mellifera scutellata* (GenBank Acc. no.: MG552701.1), *Apis mellifera intermissa* (GenBank Acc. no.: KM458618.1), and *Apis mellifera ruttneri* (GenBank Acc. no.: MN714162.1) subspecies.

#### 3.1.2. Evolutionary Origin of the Pannonian Bee in Hungary Based on the tRNAleu-Cox2 Intergenic Region (E2/H2)

A total of 124 samples were included in the analysis of the tRNAleu-cox2 (*COI-COII*) intergenic region (E2/H2) (GenBank Acc. no.: PQ724159—PQ724284). The 465 bp sequences started at base 3456 of the reference sequence (GenBank Acc. no.: MN250878.1). The sequence analysis identified six polymorphic sites (Figure S5) that were arranged on six haplotypes (Table 3). All six haplotypes, which have been previously described [24,31,60], belong to the evolutionary lineage C designated: HE1-C1 (GenBank Acc. no.: FJ824582.1), HE2-C2p (GenBank Acc. no.: HM117904), HE3-C2i (GenBank Acc. no.: JQ97703), HE4-C2d (GenBank Acc. no.: FJ824584), HE5-C2e (GenBank Acc. no.: FJ824586), and HE6-C2l (GenBank Acc. no.: GQ433625).

As for the variance of the tRNAleu-cox2 intergenic region (E2/H2), the highest haplotype diversity was found in the MF (Hd = 0.525) and REF (Hd = 0.581) groups. The nucleotide diversity was the lowest in the KRAJ group (π = 0.00054) and the highest in the REF group (π = 0.0035) (Table S8).

**Figure 2:**
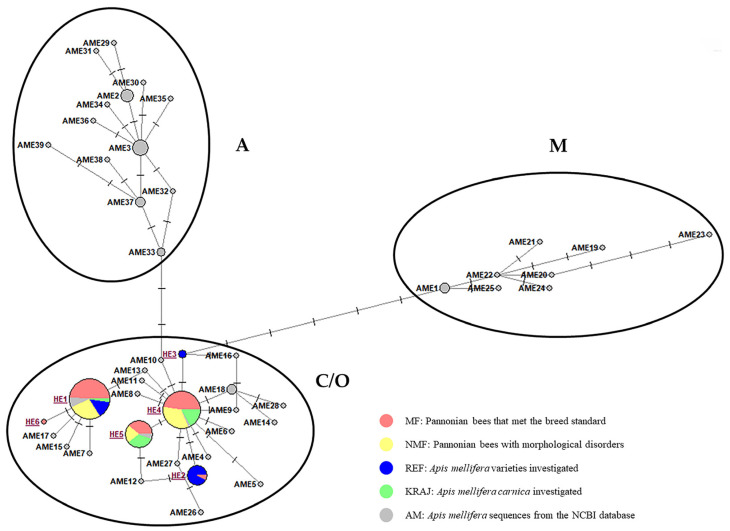
Median-joining network illustrating the relationships and the lineages of the haplotypes obtained from the tRNAleu-cox2 intergenic region (E2/H2). HE1–6—haplotypes containing our samples, AME1-39—haplotypes containing sequences of the NCBI database (Tables S7 and S9). The size of each circle is directly proportional to the frequency of the corresponding haplotypes. A, C, O and M: evolutionary lineages of honey bees. Haplotypes of reference sequences: HE1—MN250878.1, MH341407.1, AP018432.1, AP018403.1; AME1—OK075087.1, HQ260353.1, CM040891.1; AME2—MW677211.1, MN585109.1, MG552703.1, MG552697.1, MG552687.1; AME3—MW677198.1, MZ981768.1, MN585110.1, MG552699.1, MG552693.1, MG552692.1, KY614238.1; AME4—MT741505.1; AME5—MT741503.1; AME6—MT741502.1; AME7—MT741501.1; AME8—MT741500.1; AME9—MT741499.1; AME10—MT741498.1; AME11—MG788257.1; AME12—JQ977704.1; AME13—JQ973664.1; AME14—JQ973663.1; AME15—JQ754650.1; AME16—JQ754649.1; AME17—JQ754648.1; AME18—JF723978.1, KY464957.1, AP018404.1; AME19—HQ337446.1; AME20—HQ260370.1; AME21—HQ260368.1; AME22—HQ260359.1; AME23—HQ260355.1; AME24—HQ260351.1; AME25—HQ260344.1; AME26—HM117906.1; AME27—HM117905.1; HE4—MH341408.1; HE5—MW811175.1; AME28—MT188686.1; AME29—MG552701.1; AME30—MG552698.1; AME31—MG552682.1; AME32—MG552681.1; AME33—MF678581.1, KJ396182.1; AME34—KX943034.1; AME35—KX870183.1; AME36—KM458618.1; AME37—KJ396188.1, KJ396186.1, KJ396184.1; AME38—KJ396187.1; and AME39—KJ396183.1.

Most of the samples were classified into the C1 (36.29%) and C2d (33.06%) haplotypes. In the HE1 (C1) haplotype, individuals from all four groups were included, not only Pannonian and Carniolan bees but also Buckfast × Italian hybrids from the REF group as well. The HE2 (C2p) haplotype consisted of *Apis mellifera ligustica* individuals, some Buckfast hybrids, and an MF sample. Two Buckfast hybrids were classified in the HE3 (C2i) haplotype. The sequences of 124 samples studied for the tRNAlu-cox2 (*COI-COII*) intergenic region were compared with 62 from the NCBI database (Tables S7 and S9) using DnaSP v6.12.03 and Network 10 software to determine 45 haplotypes (Figure 2). The analysis separated the sequences into four evolutionary lineages: A, C, M, and O (Figure 2).

The haplotype with the largest number of individuals (49) was HE1 (C1), which included the GenBank *Apis mellifera ligustica* (GenBank Acc. no.: MH341407.1), *Apis mellifera carnica* (GenBank Acc. no.: AP018403.1, MN250878.1), Buckfast hybrid (GenBank Acc. no.: AP018432.1), and individuals of the MF, NMF, REF, and KRAJ groups.

**Figure 3:**
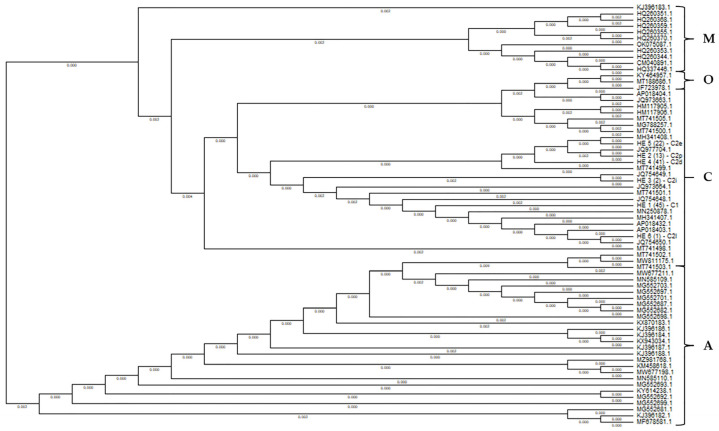
Phylogenetic analysis of the *COI-COII* intergenic region (E2/H2) for determining the evolutionary lineage of the Pannonian bee in Hungary. HE1–6—haplotypes containing our samples. The numbers in parentheses correspond to the number of individuals with a certain haplotype, which were identified using the commonly used nomenclature in the NCBI database. A, C, O and M: evolutionary lineages of honey bees.

In order to better illustrate the lineages, a phylogenetic tree was constructed (Figure 3) with the MEGA 11 software based on the same sequences as used for the network analysis (Figure 2). The tree clearly shows that the examined samples are most closely related to the haplotypes of the C evolutionary lineage, while also being relatively close to those of the evolutionary lineage O.

### 3.2. Microsatellite Marker Analysis of the Honey Bee Groups Investigated

The same 144 samples were genotyped with 20 microsatellite markers, where a total of 224 alleles were detected. The highest number of alleles was found at marker A29 (27 alleles), while the lowest was at marker Ap066 (3 alleles). The lowest mean number of alleles was found in the KRAJ group (3.7 ± 2.18), which could be attributed to the small sample size (16). The highest value for this parameter was observed in the MF group, which had the largest sample size (64) and was derived from the highest number of breeders (16) (Table 4).

The genotypes of MF were not in Hardy–Weinberg equilibrium, and this was associated with selective pressure. Heterozygosity values can be used to infer the Hardy–Weinberg equilibrium (HW), which enables the determination of the proportion of heterozygotes in the population. In two of the groups (REF, KRAJ), there was no significant deviation from HW, while significant differences were also observed in the NMF. The NMF, REF, and KRAJ had a higher proportion of heterozygotes. Our results showed that the inbreeding level is quite low for all groups, and most are close to the Hardy–Weinberg equilibrium state.

Pairwise F_ST_ comparisons were used to determine the genetic distinction between the bee groups examined (Table 5). The calculation revealed that the genetic differentiation was very low (0.008) between the MF and NMF groups, whereas the largest was between the REF and KRAJ groups (0.071). The genetic differences were significant in all cases, although less so between MF and NMF.

**Figure 4:**
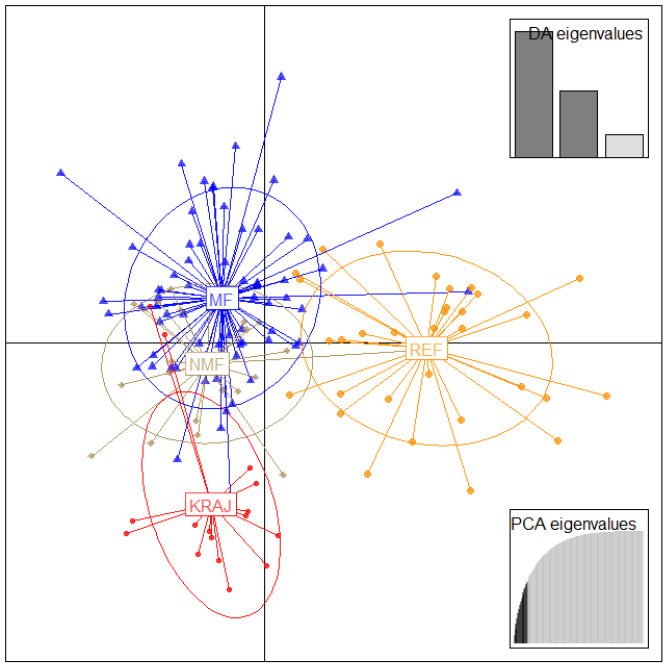
Graphical illustration of the population structure presented by Discriminant Analysis of Principal Components (DAPC). MF (blue)—Pannonian bees that met the breed standard, NMF (grey)—Pannonian bees with morphological disorders, REF (yellow)—different varieties of *Apis mellifera: ligustica, ligustica* × Buckfast hybrid, Buckfast hybrid, KRAJ (red)—*Apis mellifera carnica* from other European countries.

Discriminant Analysis of Principal Components (DAPC) showed that MF and REF were distinct, while MF and NMF partially overlapped. However, the latter two groups began to diverge genetically, with NMF becoming more similar to the Carniolan bee (Figure 4).

**Figure 5:**
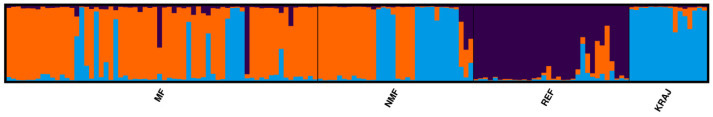
STRUCTURE clustering of the honey bee groups investigated. MF—Pannonian bees that met the breed standard, NMF—Pannonian bees with morphological disorders, REF—different varieties of *Apis mellifera: ligustica, ligustica* × Buckfast hybrid, Buckfast hybrid, KRAJ—*Apis mellifera carnica* from other European countries.

Similar results were obtained from the analysis of population structure. The cluster analysis using the STRUCTURE 2.3.4 software resulted in a most likely K-value of 3 according to the Evanno method (Figure 5 and Figure S6), showing 92 identical solutions out of 100 runs at the 97% threshold.

REF samples were split from the Pannonian (MF, NMF) and the Carniolan bees (KRAJ) clearly. The other cluster, marked with blue, was mainly present in the KRAJ group. It was also found to varying extents in the MF and NMF, with a higher proportion in the NMF group.

## 4. Discussion

Our research determined the evolutionary lineage of the native Pannonian honey bee of Hungary. We also assessed its genetic diversity and examined its relationship with *Apis mellifera carnica* from other regions of Europe, as well as other honey bee subspecies.

The mitochondrial analysis of the tRNAleu-cox2 intergenic region (E2/H2) showed that the samples investigated were in six haplotypes. All of them belonged to the C evolutionary lineage and could be identified in the NCBI database as the commonly used haplotype nomenclature (C1, C2i, C2d, C2e, C2l, and C2p). This lineage includes the North Mediterranean subspecies. The C1 haplotype has been identified in Italy, Austria, Slovenia, Hungary, Croatia, Romania, Serbia, and the USA [31,32,61]. The C2i haplotype was found in Croatia, Slovenia, Romania, Albania, Greece, Serbia, and the USA [31,32,61,62]. The C2d haplotype was present in Slovenia, Hungary, Croatia, Romania, Albania, Bulgaria, Greece, Serbia, and the USA [31,32,61,62]. The C2e haplotype was found in Croatia, Serbia, and the USA [31,32,62], while the C2l haplotype was detected in Bulgarian and Serbian populations [32,62]. The C2p haplotype was identified in the region of Romania [63].

The haplotype diversity of the Serbian and American populations belonging to the C evolutionary lineage was higher than that in the MF samples, while the nucleotide diversity was lower [61,62]. The value of the Lebanese colonies belonging to the evolutionary lineage O and the Swiss (evolutionary lineage M) populations was higher than that of the MF populations, while it was lower compared to the Guinean (evolutionary lineage A) and French (evolutionary lineage M) honey bee stocks [24].

The diversity of the *COI* gene in mitochondrial DNA resulted in amino acid substitutions. Compared to the reference sequence, the polymorphism led to amino acid substitutions in the H3 haplotype (valine to isoleucine) and the H4 haplotype (isoleucine to methionine).

In the comparison of synthetic sequences, the majority of the haplotypes were grouped together, except for H6. This haplotype included eight samples from the REF group, which showed more than 99.7% similarity to *Apis mellifera capensis* (GenBank Acc. no.: KX870183.1), *Apis mellifera iberiensis* (GenBank Acc. no.: MN585110.1), and *Apis mellifera intermissa* (GenBank Acc. no.: KM458618.1), which are haplotypes belonging to the A evolutionary lineage [28].

In the microsatellite analysis, the highest mean number of alleles was in the MF group; however, previous studies on Hungarian bees measured a higher value [36,64], which could be due to the larger sample size. In the Serbian samples, this value was higher, even though the sample size was smaller [62].

Comparing the Pannonian bee with other populations belonging to the C evolutionary lineage, the average allele number was higher in *Apis mellifera carnica* from southern and northern Serbia. The *Apis mellifera carnica* from Poland, the Buckfast hybrid from Hungary, and the *Apis mellifera ligustica* from China had lower values [64,65]. High heterozygosity levels were found in Hungarian bees in a previous study [36], whereas our results showed lower values. Southern and Northern Serbia and China had lower heterozygosity, while Poland and the Buckfast hybrid from Hungary had higher heterozygosity than our sample collection (Table 4) [64,65]. The heterozygosity of Croatian, Greek, and Italian *Apis mellifera* individuals belonging to the C evolutionary lineage was higher than the MF samples. Compared to populations of *Apis mellifera mellifera* of M evolutionary lineage (Burzyanskaya, Chastinskaya, Nytvenskaya, Osinskaya), heterozygosity values were higher in each of the groups examined in this study [66].

Based on the results of the Hardy–Weinberg test, the MF group showed a highly significant deviation, presumably due to selective pressure. In contrast, in a previous study where the colonies of the bee breeders were analyzed separately, most of the groups were close to the ideal population status [67]. However, the inbreeding coefficients were quite low (F_IS_) in our sample collection; a previous survey had an even lower level of inbreeding [36]. The MF and NMF groups were the closest by pairwise F_ST_ comparisons. This result was supported by the DAPC analysis, where individuals from these two groups partially overlapped. However, bees with morphological defects (NMF) are beginning to diverge genetically from those meeting breed standards, becoming more similar to Carniolan bees. Our results of the cluster analysis (Figure 5) showed that MF samples were more concentrated than the individuals with phenotypic disorders, as indicated by the DAPC data as well (Figure 4). The MF and REF groups were separated, and the largest genetic distance was between the REF and KRAJ groups in all analyses. Based on our results, individuals of the MF and NMF groups classified by phenotypic testing genetically differ slightly from each other. The DAPC analysis (Figure 4) showed that the Pannonian bee in Hungary (MF, NMF) has diverged from the KRAJ group as a result of natural selection (adaptation to the environment) and artificial breeding (beekeeping work, morphological traits). These findings support classifying the Pannonian honey bee as a separate breed.

In summary, we can say that according to all mitochondrial markers used in this study, the Pannonian bee of Hungary belongs to the C evolutionary lineage, which represents Northern Mediterranean honey bee subspecies. The results obtained from the microsatellite analysis suggest that the individuals of Pannonian bee colonies that strictly met the morphological breed standard show a slightly significant differentiation from those that failed the morphological breed identification. Our study may provide help in beekeeping and gene conservation, maintaining the genetic variance and preserving the genetically pure state of the selected honey bee subspecies. Moreover, a relevant sample collection could be chosen for further high-resolution marker research (SNP), in order to set up a correct marker panel for a more specific identification of the Pannonian bee.

## 5. Conclusions

This study provides important insights into the evolutionary origin and genetic diversity of the Pannonian bee, which is the only native honey bee ecotype in Hungary. Our mitochondrial DNA analysis showed that the Pannonian bee is most closely related to the subspecies in the C evolutionary lineage; however, we were able to identify several new haplotypes in native colonies. Based on the microsatellite data, our findings revealed a low level of inbreeding, with most of the bee groups studied being close to the Hardy–Weinberg equilibrium state, suggesting that proper breeding practices are being used. The molecular data highlighted a significant genetic difference between the Pannonian bee and the other subspecies. In addition, a slight divergence was observed between individuals with morphological abnormalities and those meeting breeding standards, suggesting the efficiency of selection and breeding practices. Based on our results, we can conclude that the breeding and phenotypic selection methods used in Hungary for genetic conservation of the Pannonian bee are appropriate and need to be continued to classify the Pannonian honey bee as a separate breed. The study provides valuable information for gene conservation strategies of honey bees, indicating the need for careful management of genetic resources to preserve the integrity and purity of a native breed. These results contribute to advancing beekeeping practices and supporting sustainable breeding programs in Hungary.

## Figures and Tables

**Table 1:** The distribution of individuals within the synthetic sequences generated from the *COI* and *16S* genes.

Haplotype	Number of Individuals					Position of the Polymorphism (bp)									
	Total	MF	NMF	REF	KRAJ	32	143	170	171	270	293	370	376	412	573
**H1**	106	54	19	24	9	T	C	A	G	T	T	C	A	C	T
**H2**	7				7	.	A	.	.	.	C	.	.	.	A
**H3**	1	1				.	.	G	.	.	.	.	.	.	.
**H4**	2	2				.	.	.	A	.	.	.	.	.	.
**H5**	17	4	13			.	.	.	.	.	.	T	.	.	.
**H6**	8			8		C	.	.	.	C	.	.	G	T	C

The table shows the number of polymorphic sites (total: 10) within each haplotype and the distribution of individuals within the haplotype. H1–6—haplotypes containing our samples. MF—Pannonian bees that met the breed standard, NMF—Pannonian bees with morphological disorders, REF—different varieties of *Apis mellifera: ligustica, ligustica* × Buckfast hybrid, Buckfast hybrid, KRAJ—*Apis mellifera carnica* from other European countries.

**Table 2:** Genetic variance of the honey bee groups investigated based on the joint analysis of the *COI* and *16S* mtDNA regions.

Group	H	Haplotypes	Hd ± SD	π ± SD	Fs	D	*p* (D*)
**MF**	4	H1, H3, H4, H5	0.214 ± 0.068	0.00032 ± 0.00011	−2.610	−1.29295	ns
**NMF**	2	H1, H5	0.498 ± 0.039	0.00072 ± 0.00006	1.670	1.53512	ns
**REF**	2	H1, H6	0.387 ± 0.078	0.00278 ± 0.00056	6.099	1.50012	ns
**KRAJ**	2	H1, H2	0.525 ± 0.055	0.00226 ± 0.00024	4.038	2.15124	**
**TOTAL**	6	H1, H2, H3, H4, H5, H6	0.417 ± 0.049	0.00154 ± 0.00027	0.147	−1.14724	ns

Number of haplotype (H), haplotypes included in the group (haplotypes), haplotype (Hd) and nucleotide (π) diversity with standard deviation (SD), Fs—Fu’s Fs value, D—Tajima’s D value and their significance level (*p* (D*)) calculated by DnaSP v6.12.03 software. ns = non-significant ** *p* ≤ 0.05. MF—Pannonian bees that met the breed standard, NMF—Pannonian bees with morphological disorders, REF—different varieties of *Apis mellifera*: *ligustica*, *ligustica* × Buckfast hybrid, Buckfast hybrid, KRAJ—*Apis mellifera carnica* from other European countries.

**Table 3:** The distribution of individuals within the tRNAleu-cox2 intergenic region (E2/H2).

Haplotype	Number of Individuals					Position of the Polymorphism (bp)					
	Total	MF	NMF	REF	KRAJ	17	61	113	134	179	314
**HE1 (C1)**	45	24	13	7	1	.	.	.	.	T	C
**HE2 (C2p)**	13	1		12		.	A	.	.	.	.
**HE3 (C2i)**	2			2		.	.	.	A	.	.
**HE4 (C2d)**	41	20	14		7	T	T	A	G	C	T
**HE5 (C2e)**	22	9	5		8	-	.	.	.	.	.
**HE6 (C2l)**	1	1				.	.	-	.	.	.

The table shows the number of polymorphism sites within each haplotype (total: 6) and the distribution of individuals within the haplotype. MF—Pannonian bees that met the breed standard, NMF—Pannonian bees with morphological disorders, REF—different varieties of *Apis mellifera: ligustica, ligustica* × Buckfast hybrid, Buckfast hybrid, KRAJ—*Apis mellifera carnica* from other European countries. The haplotype names in brackets correspond to the commonly accepted nomenclature in the NCBI database.

**Table 4:** Standard diversity parameters for all honey bee groups investigated across 20 microsatellite loci.

Group	Sample Size	MNA ± SD	H_E_ ± SD	H_O_ ± SD	F_IS_	HW
**MF**	64	9.3 ± 4.93	0.59 ± 0.046	0.54 ± 0.014	0.088	***
**NMF**	32	6.5 ± 3.27	0.55 ± 0.052	0.52 ± 0.020	0.053	*
**REF**	32	7.2 ± 3.51	0.64 ± 0.051	0.62 ± 0.019	0.029	ns
**KRAJ**	16	3.7 ± 2.18	0.47 ± 0.054	0.47 ± 0.028	0.013	ns

ns = non-significant, * *p* < 0.05, *** *p* < 0.001. Mean number of alleles (MNA), unbiased expected (H_E_) and observed (H_O_) heterozygosity with standard deviation (SD), inbreeding coefficient (F_IS_), deviation from the Hardy–Weinberg equilibrium (HW). MF—Pannonian bees that met the breed standard, NMF—Pannonian bees with morphological disorders, REF—different varieties of *Apis mellifera: ligustica, ligustica* × Buckfast hybrid, Buckfast hybrid, KRAJ—*Apis mellifera carnica* from other European countries.

**Table 5:** Pairwise F_ST_ estimates and their significance level between the honey bee groups investigated.

	MF	NMF	REF	KRAJ
**MF**		0.005	0.000	0.000
**NMF**	0.008		0.000	0.000
**REF**	0.042	0.047		0.000
**KRAJ**	0.032	0.037	0.071	

Pairwise F_ST_ values are below the diagonal, and the probability, *p* (rand ≥ data), based on 9999 permutations, is shown above the diagonal, calculated by AMOVA. MF—Pannonian bees that met the breed standard, NMF—Pannonian bees with morphological disorders, REF—different varieties of *Apis mellifera: ligustica, ligustica* × Buckfast hybrid, Buckfast hybrid, KRAJ—*Apis mellifera carnica* from other European countries.

## Data Availability

The original contributions presented in this study are included in the article/Supplementary Material. Further inquiries can be directed to the corresponding author.

## Supplementary Materials

The following supporting information can be downloaded at: https://www.mdpi.com/article/10.3390/biology14050475/s1, Figure S1: Representative chromatograms for the *COI* sequences; Figure S2: Amino acid substitution resulting from a polymorphism at position 180 in the *COI* gene; Figure S3: Amino acid substitution resulting from a polymorphism at position 181 in the *COI* gene; Figure S4: Representative chromatograms for the *16S* sequences; Figure S5: Representative chromatograms for the E2/H2 sequences; Figure S6: Calculation of the most likely clustering based on the Evanno method; Table S1: Sample collection by apiaries and colonies; Table S2: Microsatellite marker sets, their chromosome position and characteristics used for PCR and capillary gel electrophoresis; Table S3: The distribution of individuals within the *COI* gene; Table S4: The distribution of individuals within the *16S* gene; Table S5: Genetic variance of the honey bee groups investigated based on the *COI* mtDNA region; Table S6: Genetic variance of the honey bee groups investigated based on the *16S* mtDNA region; Table S7: Sequences of different *Apis mellifera* subspecies from the NCBI database used for the mitochondrial DNA analysis; Table S8: Genetic variance of the honey bee groups investigated based on the *COI-COII* (E2/H2) intergenic mtDNA region; Table S9: Haplotype sequences with their commonly accepted nomenclature from the NCBI database used for the evaluation of *COI-COII* intergenic region (E2/H2).
