# Supplementary material for: Evolutionary Origin and Genetic Diversity of the Pannonian Ecotype of Apis mellifera carnica Colonies in Hungary Based on Mitochondrial DNA and Microsatellite Markers
Source: Biology (Basel). 2025 Apr 25;14(5):475. doi: 10.3390/biology14050475 (PMC12108602; doi:10.3390/biology14050475)
Supplement: Supplementary file 1 [file biology-14-00475-s001.zip › biology-3524674-supplementary.pdf]

Figure S1. Representative chromatograms for the *COI* sequences.

Figure S2. Amino acid substitution resulting from a polymorphism at position 180 in the *COI* gene.

Figure S3. Amino acid substitution resulting from a polymorphism at position 181 in the *COI* gene.

Figure S4. Representative chromatograms for the *16S* sequences.

Figure S5. Representative chromatograms for the E2/H2 sequences.

Figure S6. Calculation of the most likely clustering based on the Evanno method.

Table S1. Sample collection by apiaries and colonies.

Table S2. Microsatellite marker sets, their chromosome position and characteristics used for PCR and capillary gel electrophoresis.

Table S3. The distribution of individuals within the *COI* gene.

Table S4. The distribution of individuals within the *16S* gene.

Table S5. Genetic variance of the honey bee groups investigated based on the *COI* mtDNA region.

Table S6. Genetic variance of the honey bee groups investigated based on the *16S* mtDNA region.

Table S7. Sequences of different *Apis mellifera* subspecies from the NCBI database used for the mitochondrial DNA analysis.

Table S8. Genetic variance of the honey bee groups investigated based on the *COI-COII* (E2/H2) intergenic mtDNA region.

Table S9. Haplotype sequences with their commonly accepted nomenclature from the NCBI database used for the evaluation of *COI-COII* intergenic region (E2/H2).

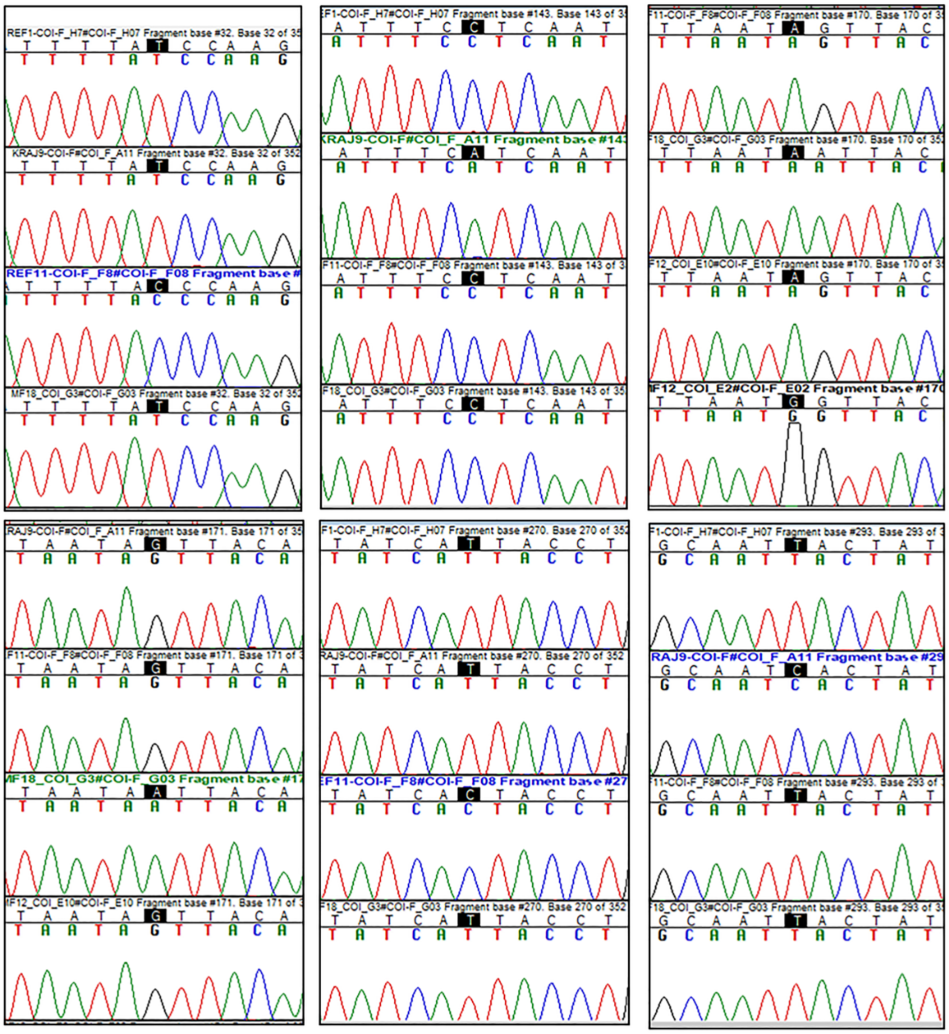

Figure S1. Representative chromatograms for the *COI* sequences.

Score	Expect	Method	Identities	Positives	Gaps
434 bits(1116)	3e-161	Compositional matrix adjust.	241/244(99%)	242/244(99%)	2/244(0%)

```
Query  1    IIKSTOPFISTNHKNIGILYIILALSTOPSGILGSSMETRLIIRIELRSPGSSTOPINND  60
            IIKSTOPFISTNHKNIGILYIILALSTOPSGILGSSMETRLIIRIELRSPGSSTOPINND
Sbjct  1    IIKSTOPFISTNHKNIGILYIILALSTOPSGILGSSMETRLIIRIELRSPGSSTOPINND  60

Query  61   QIYNTIVTSHAFLIIFFIVIPFLIGGFGNWLIPLILGSPDIAFPRINNIRFSTOPLLPPS  120
            QIYNTIVTSHAFLIIFFIVIPFLIGGFGNWLIPLILGSPDIAFPRINNIRFSTOPLLPPS
Sbjct  61   QIYNTIVTSHAFLIIFFIVIPFLIGGFGNWLIPLILGSPDIAFPRINNIRFSTOPLLPPS  120

Query  121  LFILLLRNLFYPRPGTGSTOPTVYPPLSAYLYHSSPSVDFAIFSLHISGISSIIGSLNLI  180
            LFILLLRNLFYPRPGTGSTOPTVYPPLSAYLYHSSPSVDFAIFSLHISGISSIIGSLNL+
Sbjct  121  LFILLLRNLFYPRPGTGSTOPTVYPPLSAYLYHSSPSVDFAIFSLHISGISSIIGSLNLM  180

Query  181  --VTIIIIIKNFSINYDQISLFPSTOPSVFITAILLIISLPVLAGAITILLFDRNFNTSFF  238
              VTIIIIIKNFSINYDQISLFPSTOPSVFITAILLIISLPVLAGAITILLFDRNFNTSFF
Sbjct  181  ETVTIIIIIKNFSINYDQISLFPSTOPSVFITAILLIISLPVLAGAITILLFDRNFNTSFF  240

Query  239  DPIG  242
            DPIG
Sbjct  241  DPIG  244
```

Figure S2. Amino acid substitution resulting from a polymorphism at position 180 in the *COI* gene.

Score	Expect	Method	Identities	Positives	Gaps
438 bits(1127)	4e-163	Compositional matrix adjust.	241/242(99%)	242/242(100%)	0/242(0%)

```
Query  1    IIKSTOPFISTNHKNIGILYIILALSTOPSGILGSSMETRLIIRIELRSPGSSTOPINND  60
            IIKSTOPFISTNHKNIGILYIILALSTOPSGILGSSMETRLIIRIELRSPGSSTOPINND
Sbjct  1    IIKSTOPFISTNHKNIGILYIILALSTOPSGILGSSMETRLIIRIELRSPGSSTOPINND  60

Query  61   QIYNTIVTSHAFLIIFFIVIPFLIGGFGNWLIPLILGSPDIAFPRINNIRFSTOPLLPPS  120
            QIYNTIVTSHAFLIIFFIVIPFLIGGFGNWLIPLILGSPDIAFPRINNIRFSTOPLLPPS
Sbjct  61   QIYNTIVTSHAFLIIFFIVIPFLIGGFGNWLIPLILGSPDIAFPRINNIRFSTOPLLPPS  120

Query  121  LFILLLRNLFYPRPGTGSTOPTVYPPLSAYLYHSSPSVDFAIFSLHISGISSIIGSLNLI  180
            LFILLLRNLFYPRPGTGSTOPTVYPPLSAYLYHSSPSVDFAIFSLHISGISSIIGSLNLI
Sbjct  121  LFILLLRNLFYPRPGTGSTOPTVYPPLSAYLYHSSPSVDFAIFSLHISGISSIIGSLNLI  180

Query  181  VTIIIIIKNFSINYDQISLFPSTOPSVFITAILLIISLPVLAGAITILLFDRNFNTSFFDP  240
            +TIIIIIKNFSINYDQISLFPSTOPSVFITAILLIISLPVLAGAITILLFDRNFNTSFFDP
Sbjct  181  ITIIIIIKNFSINYDQISLFPSTOPSVFITAILLIISLPVLAGAITILLFDRNFNTSFFDP  240

Query  241  IG  242
            IG
Sbjct  241  IG  242
```

Figure S3. Amino acid substitution resulting from a polymorphism at position 181 in the *COI* gene.

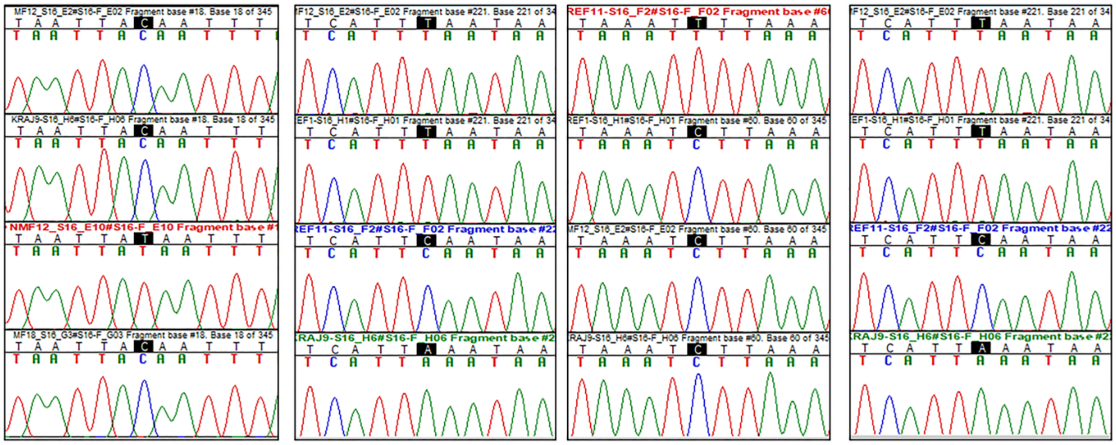

Figure S4. Representative chromatograms for the *16S* sequences.

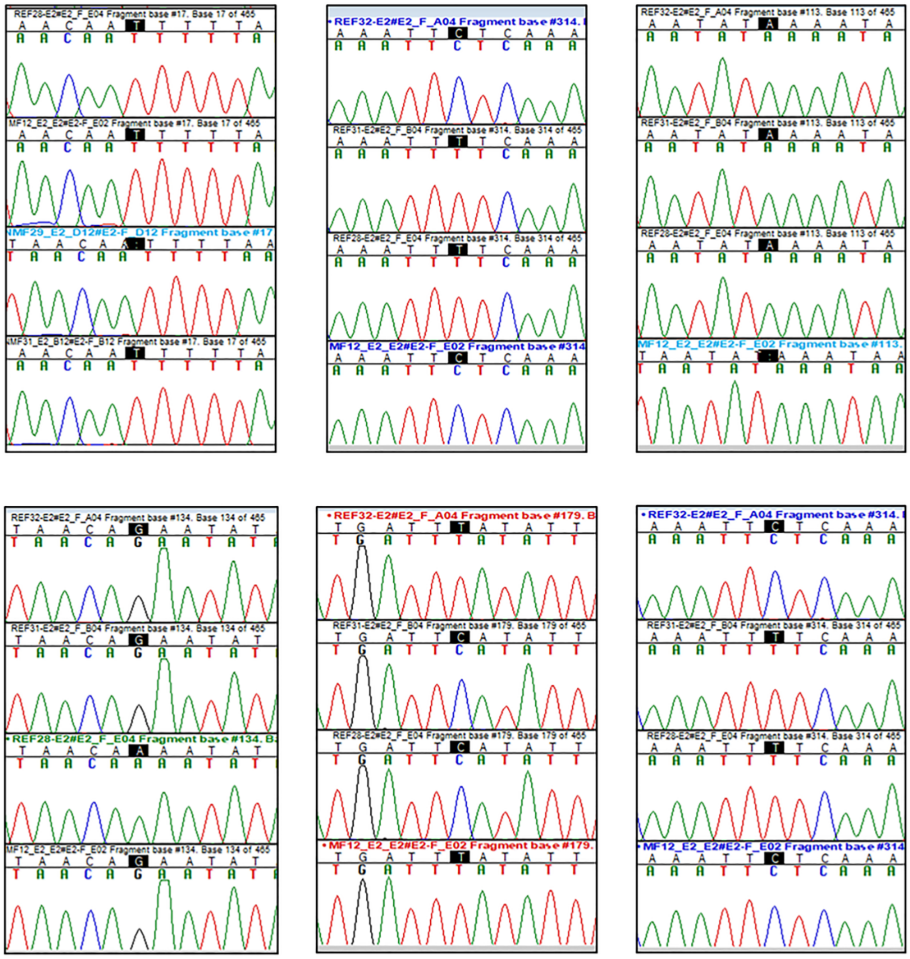

Figure S5. Representative chromatograms for the E2/H2 sequences.

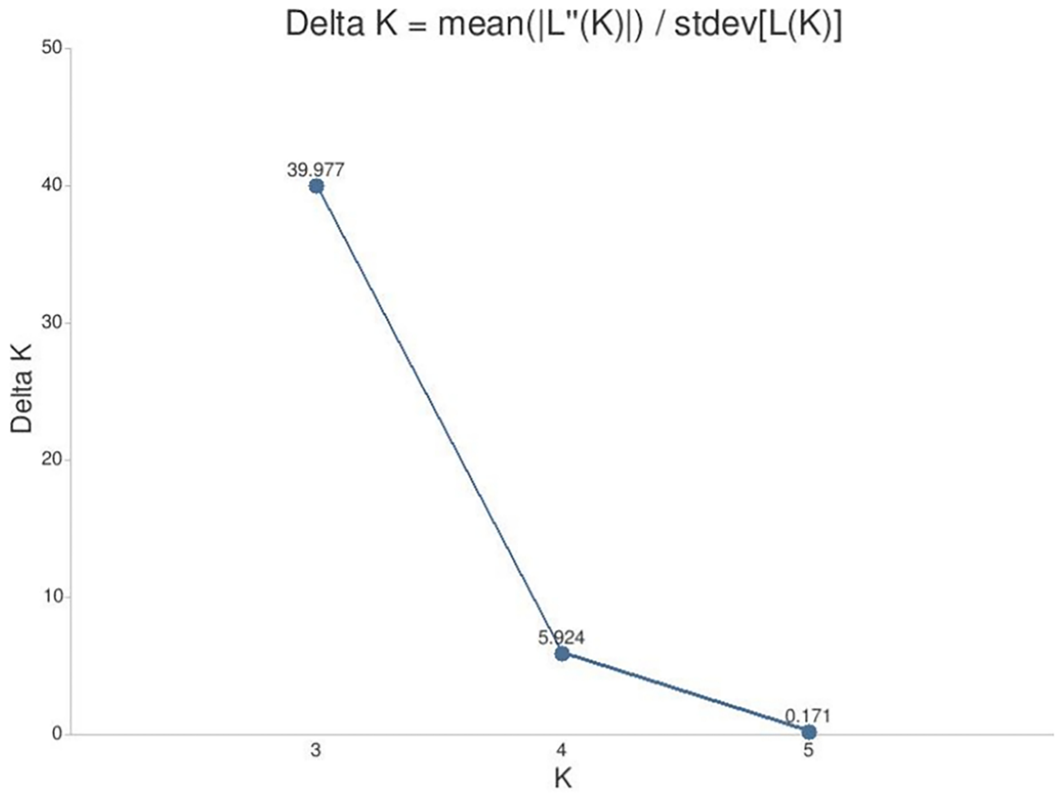

Figure S6. Calculation of the most likely clustering based on the Evanno method.

Table S1. Sample collection by apiaries and colonies.

	Total samples	Apiaries	Colonies/ apiary	Samples/ colony
**MF**	64	16	4	1
**NMF**	32	8	1	4
**REF**	32	4	2	4
**KRAJ**	16	2	1	8

The table shows the number of breeders and colonies from where the samples were collected, as well as the number of individuals selected from each colony.

MF - Pannonian bees that met the breed standard, NMF - Pannonian bees with morphological disorders, REF - different varieties of *Apis mellifera*: *ligustica, ligustica* x Buckfast hybrid, Buckfast hybrid, KRAJ - *Apis mellifera carnica* from other European countries.

Table S2. Microsatellite marker sets, their chromosome position and characteristics used for PCR and capillary gel electrophoresis.

Fragment analysis	Multiplex PCR	Microsatellite marker	Chromosome	Position (bp)	Allele size with tail sequence (bp)	Fluorescent labelling (WELL-RED)	Tm (°C)	Cycle number
**FA1**	M1	Ap066	3	4,186,880-4,186,980	101-133	D2	60	30
		Ac306	2	7,639,260-7,639,424	175-211			
	M5	Ap218	5	6,946,374-6,946,491	132-149	D4	60	30
		A29	15	14,619-14,756	153-210			
		Ap049	2	8,918,415-8,918,556	140-174	D3	60	30
		Ap033	10	8,690,103-8,690,339	239-275	D3	60	30
**FA2**	M2	A007	8	2,799,436-2,799,558	123-158	D2	60	30
		A107	7	6,998,252-6,998,427	160-194			
	M3	Ac011	9	410,642-410,766	125-149	D3	60	30
		Ap043	3	868,728-868,902	151-189			
	M6	Ap081	9	2,819,248-2,819,375	147-161	D4	60	30
		Ap055	1	24,902,569-24,902,762	181-227			
		Ap226	1	19,714,963-19,715,197	249-263			
**FA3**	M7	A(B)24	7	2,236,580-2,236,674	112-124	D3	57	35
		A113	6	6,380,984-6,381,203	216-256			
	M8	A35	1	1,983,189-1,983,288	117-142	D2	57	35
		A88	unknown	-	157-172			
		Ap289	8	7,410,240-7,410,438	193-234			
		Ap307	16	2,748,961-2,749,109	158-166	D4	60	30
		A008	2	4,309,399-4,309,574	182-200	D3	60	30

Table S3. The distribution of individuals within the *COI* gene.

COI	Sequence	Number of individuals				
**Haplotype**		**Total**	**MF**	**NMF**	**REF**	**KRAJ**
**HC1***	TCAGTTC	124	59	32	24	9
**HC2***	C...C..	8			8	
**HC3***	...A…	2	2			
**HC4***	..G….	1	1			
**HC5***	.A...C.	7				7

The table shows the number of polymorphic sites (total: 7) within each haplotype and the distribution of individuals within the haplotype.

* new haplotype

ns = non-significant, *P<0.05, **P<0.01, ***P<0.001

HC1-5 - haplotypes containing our samples

MF - Pannonian bees that met the breed standard, NMF - Pannonian bees with morphological disorders, REF - different varieties of *Apis mellifera*: *ligustica*, *ligustica* x Buckfast hybrid, Buckfast hybrid, KRAJ - *Apis mellifera carnica* from other European countries.

Table S4. The distribution of individuals within the *16S* gene.

16S	Sequence	Number of individuals				
**Haplotype**		**Total**	**MF**	**NMF**	**REF**	**KRAJ**
**HS1**	CACT	109	57	19	24	9
**HS2**	.GTC	8			8	
**HS3***	T…	17	4	13		
**HS4***	...A	7				7

The table shows the number of polymorphic sites (total: 4) within each haplotype and the distribution of individuals within the haplotype.

* new haplotype

HS1-4 - haplotypes containing our samples

MF - Pannonian bees that met the breed standard, NMF - Pannonian bees with morphological disorders, REF - different varieties of *Apis mellifera*: *ligustica*, *ligustica* x

Buckfast hybrid, Buckfast hybrid, KRAJ - *Apis mellifera carnica* from other European countries.

Table S5. Genetic variance of the honey bee groups investigated based on the *COI* mtDNA region.

Group	H	Hd ± SD	π ± SD	Fs	D	p (D*)
**MF**	3	0.095 ± 0.050	0.00027 ± 0.00015	-2.607	-1.31538	ns
**NMF**	1	0	0	0	0	ns
**REF**	2	0.387 ± 0.078	0.0022 ± 0.00045	2.693	1.12316	ns
**KRAJ**	2	0.525 ± 0.055	0.00298 ± 0.00031	2.834	1.89943	**
**TOTAL**	5	0.233 ± 0.046	0.0012 ± 0.00027	-1.551	-1.25625	ns

Number of haplotype (H), haplotype (Hd) and nucleotide (π) diversity with standard deviation (SD), Fs – Fu's Fs value and D – Tajima's D value.

ns = non-significant (P > 0.10), *0.05 < P ≤ 0.10, **P ≤ 0.05

MF - Pannonian bees that met the breed standard, NMF - Pannonian bees with morphological disorders, REF - different varieties of *Apis mellifera*: *ligustica*, *ligustica* x Buckfast hybrid, Buckfast hybrid, KRAJ - *Apis mellifera carnica* from other European countries.

Table S6. Genetic variance of the honey bee groups investigated based on the *16S* mtDNA region.

Group	H	Hd ± SD	π ± SD	Fs	D	p (D*)
**MF**	2	0.125 ± 0.055	0.00036 ± 0.00016	-0.261	-0.53298	ns
**NMF**	2	0.498 ± 0.039	0.00144 ± 0.00011	1.67	1.53512	ns
**REF**	2	0.387 ± 0.078	0.00337 ± 0.00068	3.932	1.29187	ns
**KRAJ**	2	0.525 ± 0.055	0.00152 ± 0.00016	1.333	1.4737	ns
**TOTAL**	4	0.385 ± 0.048	0.0018 ± 0.00031	0.367	-0.62152	ns

Number of haplotypes (H), haplotype (Hd) and nucleotide (π) diversity with standard deviation (SD), Fs – Fu's Fs value and D – Tajima's D value.

ns = non-significant (P > 0.10), *0.05 < P ≤ 0.10, **P ≤ 0.05

MF - Pannonian bees that met the breed standard, NMF - Pannonian bees with morphological disorders, REF - different varieties of *Apis mellifera*: *ligustica*, *ligustica* x

Buckfast hybrid, Buckfast hybrid, KRAJ - *Apis mellifera carnica* from other European countries.

Table S7. Sequences of different *Apis mellifera* subspecies from the NCBI database used for the mitochondrial DNA analysis.

	**GenBank accession number**	***Apis mellifera* subspecies**
**1.**	AP018403.1	*carpatica*
**2.**	AP018404.1	*caucasica*
**3.**	AP018432.1	*buckfast*
**4.**	CM040891.1	*mellifera*
**5.**	KJ396182.1	*mellifera*
**6.**	KJ396183.1	*mellifera*
**7.**	KJ396184.1	*mellifera*
**8.**	KJ396185.1	*mellifera*
**9.**	KJ396186.1	*mellifera*
**10.**	KJ396187.1	*mellifera*
**11.**	KJ396188.1	*mellifera*
**12.**	KJ396189.1	*mellifera*
**13.**	KM458618.1	*intermissa*
**14.**	KP163643.1	*syriaca*
**15.**	KX870183.1	*capensis*
**16.**	KX943034.1	*scutellata x capensis*
**17.**	KY464957.1	*meda*
**18.**	KY464958.1	*lamarckii*
**19.**	KY614238.1	*scutellata*
**20.**	KY926884.1	*mellifera*
**21.**	MF678581.1	*monticola*
**22.**	MG552681.1	*capensis*
**23.**	MG552682.1	*capensis*
**24.**	MG552687.1	*capensis*
**25.**	MG552692.1	*capensis*
**26.**	MG552693.1	*capensis*
**27.**	MG552694.1	*capensis*
**28.**	MG552697.1	*capensis x scutellata*
**29.**	MG552698.1	*scutellata*
**30.**	MG552699.1	*scutellata*
**31.**	MG552701.1	*scutellata*
**32.**	MG552703.1	*scutellata*
**33.**	MH341407.1	*ligustica*

**34.**	MH341408.1	*ligustica*
**35.**	MN119925.1	*unicolor*
**36.**	MN250878.1	*carnica*
**37.**	MN585108.1	*simensis*
**38.**	MN585109.1	*adansonii*
**39.**	MN585110.1	*iberiensis*
**40.**	MN714161.1	*jemenitica*
**41.**	MN714162.1	*ruttneri*
**42.**	MT188686.1	*anatoliaca*
**43.**	MW811175.1 (=NC_061380)	*carnica*
**44.**	MZ981768.1	*siciliana*
**45.**	OK075087.1	*iberiensis*

Table S8. Genetic variance of the honey bee groups investigated based on the *COI-COII* (E2/H2) intergenic mtDNA region.

**Group**	**H**	**Haplotypes**	**Hd ± SD**	**π ± SD**	**Fs**	**D**	**p (D*)**
**MF**	5	HE1, HE2, HE4, HE5, HE6	0.525 ± 0.023	0.00226 ± 0.00010	2.325	1.2053	ns
**NMF**	3	HE1, HE4, HE5	0.498 ± 0.039	0.00215 ± 0.00017	3.421	2.02053	*
**REF**	3	HE1, HE2, HE3	0.581 ± 0.075	0.0035 ± 0.00044	2.606	1.32453	ns
**KRAJ**	3	HE1, HE4, HE5	0.125 ± 0.106	0.00054 ± 0.00046	0.177	-1.49796	ns
**TOTAL**	4	HE1, HE2, HE3, HE4, HE5, HE6	0.598 ± 0.024	0.00251 ± 0.00012	2.167	1.08468	ns

Number of haplotypes (H), haplotypes included in the group (Haplotypes), haplotype (Hd) and nucleotide (π) diversity with standard deviation (SD), Fs – Fu's Fs value and D – Tajima's D value.

ns = non-significant ($P > 0.10$), *$0.05 < P \leq 0.10$, **$P \leq 0.05$

MF - Pannonian bees that met the breed standard, NMF - Pannonian bees with morphological disorders, REF - different varieties of *Apis mellifera*: *ligustica*, *ligustica* x Buckfast hybrid, Buckfast hybrid, KRAJ - *Apis mellifera carnica* from other European countries.

Table S9. Haplotype sequences with their commonly accepted nomenclature from the NCBI database used for the evaluation of *COI-COII* intergenic region (E2/H2).

	**GenBank accession number**	**Haplotype names**
**1.**	FJ824582.1	C1
**2.**	FJ824584.1	C2d
**3.**	FJ824586.1	C2e
**4.**	GQ433625.1	C2l
**5.**	HM117904.1	C2p
**6.**	HM117905.1	C2q
**7.**	HM117906.1	C2r
**8.**	HQ260344.1	M17a
**9.**	HQ260351.1	M40
**10.**	HQ260353.1	M42
**11.**	HQ260355.1	M46
**12.**	HQ260359.1	M50
**13.**	HQ260368.1	M24
**14.**	HQ260370.1	M19a
**15.**	HQ337446.1	M13
**16.**	JF723978.1	C2j
**17.**	JQ754648.1	C2z
**18.**	JQ754649.1	C2x
**19.**	JQ754650.1	C2y
**20.**	JQ973663.1	C2t
**21.**	JQ973664.1	C2v
**22.**	JQ97703.1	C2i
**23.**	JQ977704.1	C2o
**24.**	MG788257.1	C2aa
**25.**	MT741498.1	C2u
**26.**	MT741499.1	C2w
**27.**	MT741500.1	C2ab
**28.**	MT741501.1	C2ac
**29.**	MT741502.1	C2ad
**30.**	MT741503.1	C2ae
**31.**	MT741505.1	C2ag
**32.**	MW677198.1	A1e
**33.**	MW677211.1	A4p
